# Influence of the cranial base flexion on Class I, II and III malocclusions: a systematic review

**DOI:** 10.1590/2177-6709.22.5.056-066.oar

**Published:** 2017

**Authors:** Kélei Cristina Mathias de Almeida, Taísa Boamorte Raveli, Camila Ivini Viana Vieira, Ary dos Santos-Pinto, Dirceu Barnabé Raveli

**Affiliations:** 1 Universidade Estadual Paulista, Faculdade de Odontologia de Araraquara, Departamento de Ortodontia e Odontopediatria (Araraquara/SP, Brazil).; 2 Private practice (Araraquara/SP, Brazil).

**Keywords:** Malocclusion, Cephalometry, Growth, Skull base

## Abstract

**Objective:**

The aim of this study was to perform a systematic review on the morphological characteristics of the skull base (flexion, anterior length and posterior length) and the concomitant development of malocclusions, by comparing differences in dimorphism, ethnicity and age.

**Methods:**

The articles were selected by means of electronic search on BBO, MEDLINE and LILACS databases from 1966 to 2016. A qualitative evaluation of the methodologies used on the articles was also performed.

**Results:**

Although the literature on this topic is abundant, only 16 articles were selected for the present systematic review. The cranial base angle itself does not seem to play a significant role in the development of malocclusions. In fact, the cranial base angle is relatively stable at the ages of 5 to 15 years.

**Conclusions:**

A more obtuse angle at the skull base, in association or not with a greater anterior length of the cranial base, can contribute to the development of Class II division 1 malocclusions. On the other hand, a more acute angle at the skull base can contribute to a more anterior positioning of the mandible and to the development of Class III malocclusions.

## RESUMO

**Objetivo:**

o objetivo desse estudo foi realizar uma revisão sistemática sobre as características morfológicas da base do crânio (flexão, comprimento anterior e comprimento posterior) e o desenvolvimento concomitante da má oclusão, comparando as diferenças do dimorfismo, etnia e idade.

**Métodos:**

os artigos foram selecionados por meio de busca eletrônica nas bases de dados BBO, MEDLINE e LILACS, de 1966 a 2016. Uma avaliação qualitativa da metodologia dos artigos também foi executada.

**Resultados:**

ainda que a literatura seja abundante nesse assunto, somente 16 artigos foram selecionados para a presente revisão sistemática. O ângulo da base do crânio, por si só, não parece desempenhar papel significativo no desenvolvimento das más oclusões. De fato, o ângulo da base do crânio é relativamente estável dos 5 aos 15 anos.

**Conclusões:**

um ângulo mais obtuso na base do crânio, associado ou não a um comprimento maior, pode contribuir para o desenvolvimento da má oclusão de Classe II, divisão 1. Por outro lado, um ângulo mais agudo na base do crânio pode contribuir para um posicionamento mais anterior da mandíbula e para o desenvolvimento da má oclusão de Classe III.

## INTRODUCTION

The skull base plays a key role in the craniofacial growth, thus helping to integrate different growth patterns both spatially and functionally, regarding several regions adjacent to the skull - such as brain components, nasal cavity, oral cavity and pharynx.^1^ In this way, the skull base supports the brain and allows the neurocranium and viscerocranium to adapt and develop during growth.^2^ ^,^ ^3^ It is reported that the first growth spurt of the skull base occurs between 14 and 32 weeks of intra-uterine life, and the second one occurs during the first year of life.^4^

The development of the skull base is closely related to both middle region of the face and mandibular positioning, with its anterior-posterior growth playing an important role in mandibular and nasomaxillary growth, thus directly contributing to the degree of facial prognathism.^5^ Based on geometrical relationships, it would be reasonable to say that any change in the skull base flexion might affect the positioning of maxilla and mandible, thus influencing skeletal pattern and occlusion as well.^6^

The literature is abundant, but controversial, regarding the influence of the skull base flexion on the development of malocclusions.^6^ Although there are studies supporting that the skull base flexion is not a factor in the etiology of malocclusions, others suggest the contrary.^2^ ^,^ ^7^ In fact, several authors^2^ ^,^ ^8^ ^-^ ^12^ showed evidence that the skull base has a considerable influence in the inter-maxillary relationships.

Ricketts^9^ reported that the skull base area has an important influence on total facial prognathism and development of anteroposterior relationship between maxilla and mandible. According to the same author, the Class II malocclusion worsens with age. To Hopkin et al,^10^ the skull base angle was lower in individuals with Class III malocclusions (males = 122.4^o^ and females = 122.2^o^) and higher in those with Class II division 1 (males = 126.7^o^ and females = 128.8^o^). By analyzing the craniofacial relationships in the mandibular prognathism and comparing them to Class I malocclusions, Horowitz and Converse^11^ found mean values of 119.3^o^ for the BaSN angle in Class III malocclusions with horizontal growth pattern, 116.6^o^ in Class III malocclusions with vertical growth pattern, and 124.1^o^ in Class I malocclusions.

Among studies showing no influence of the skull base on malocclusions, Hildwein et al^13^ found no significant difference in the BaSN angle in individuals with Class II and Class I malocclusions. Kasai et al^14^ investigated the relationship between skull base and maxillofacial morphology in Japanese subjects and found no difference between Class I and Class II malocclusions. Similarly, Wilhelm et al^15^ observed no difference in the measurements of the skull base regarding Class I and Class II malocclusions.^1^

There are many studies corroborating the finding that skull base flexion has an influence on malocclusions, whereas others show no such evidence. Different factors can contribute to these divergent findings, such mixed samples - in terms of age and gender - as well as the use of chronological age instead of skeletal age. Other factors possibly contributing to these divergent findings include the following: lack of radiographic standardization, small sample size, and number of inter-group comparisons. In this way, the influence of the skull base flexion as an etiological factor influencing inter-maxillary relationships is still a matter of debate and investigation.^16^

The objective of the present study was to perform a systematic review on the relationship between skull base (flexion, anterior length and posterior length) and the development of malocclusions, by comparing differences in gender dimorphism, ethnicity and age.

## METHODS 

### Search strategy

The articles selected for this systematic review of the literature were found by means of electronic search on BBO, MEDLINE and LILACS databases, from 1966 to 2016. The keywords used for this bibliographic search were the following: skull base, cephalometry, malocclusion, Class I malocclusion, Class II malocclusion, Class III malocclusion.

Next, a manual search was also performed by analyzing the bibliographic references of the articles according to the systematic review.

### Criteria for study selection

The inclusion criteria were the following:

» Studies using lateral cephalometric radiography.
» Meta-analysis, randomized clinical studies, retrospective and prospective studies.
» Studies published from 1966 to 2016.
» Studies addressing non-treated Class I, Class II and Class II malocclusions.
» Studies with subjects aged between 6 and 12 years old (mixed dentition) and those aged between 12 to 18 years old (permanent dentition).
» Studies on skull base using linear (S-N, S-Ar, S-Ba) or angular (NSAr and NsBa) measurements.
» Articles written in Portuguese, English or Spanish idioms.

The exclusion criteria were the following:

» Clinical cases, descriptive studies, opinion articles or abstracts.
» Case studies.
» Adult studies (subjects over 18 years old).
» Animal studies or laboratory studies.
» Craniofacial syndromes.
» Dissertations.

### Article selection

Four researchers have independently examined titles, keywords and abstracts of the articles found in the databases, according to the inclusion and exclusion criteria aforementioned. The articles were consensually selected and integrally considered, and after reading them a final decision was made regarding their inclusion or not in the present study. The articles were classified according to the criteria summarized in Table 1.

**Table 1:** Qualification of the methodology used by the articles selected for review.

	2 points	1 points
Clearly formulated objective	(x) yes	( ) no
Study design	(x) yes	( ) no
Description of the sample selection	(x) yes	( ) no
Adequate sample size	(x) yes	( ) no
Control group	(x) yes	( ) no
Adequate measurement method	(x) yes	( ) no
Adequate statistical analysis	(x) yes	( ) no
Method error analysis	(x) yes	( ) no
Blind study	(x) yes	( ) no
Multiple comparison between Class I, II and III malocclusions	(x) yes	( ) no
Total		20 points

## RESULTS

A total of 315 articles were initially identified, 8 from BBO, 12 from LILACS and 295 from MEDLINE. After reading the abstracts, only 55 were selected: no article from BBO, 2 from LILACS and 53 from MEDLINE. These were fully considered and after applying the inclusion and exclusion criteria, 39 articles were excluded and 16 remained, all from the MEDLINE database (Table 2). Next, another article was excluded due to lack of the measurements considered for review.

**Table 2:** Evaluation of methodological quality of the 16 articles selected for review.

Article	Clear objective	Study design	Description of the sample selection	Adequate sample size	Control group	Adequate measurement method	Adequate statistical analysis	Method error analysis	Blind study	Multiple comparison between Class I, II and III malocclusions	Score
Dhoptkar et al.^19^	Yes	No	Yes	Yes	No	Yes	Yes	Yes	No	Yes	17
Wilhelm et al.^15^	Yes	Yes	Yes	Yes	No	Yes	Yes	No	No	No	16
Mouakeh^21^	Yes	No	Yes	Yes	Yes	Yes	Yes	No	No	No	16
Alexander et al^22^	Yes	Yes	Yes	Yes	No	Yes	Yes	Yes	No	No	17
Ishii et al.^23^	Yes	No	No	Yes	Yes	Yes	Yes	Yes	No	No	16
Polat, Kaya^1^	Yes	No	Yes	Yes	No	Yes	Yes	Yes	No	Yes	17
Singh et al^24^	Yes	No	Yes	Yes	Yes	Yes	Yes	No	No	No	16
Johannsdottir et al.^25^	Yes	No	Yes	Yes	Yes	Yes	Yes	No	No	No	16
Rothstein, Yoon-Tarlie^16^	Yes	No	Yes	Yes	Yes	Yes	Yes	No	No	No	16
Ishii et al.^26^	Yes	No	Yes	Yes	Yes	Yes	Yes	No	No	No	16
Kappor et al.^27^	Yes	Yes	Yes	Yes	Yes	Yes	Yes	No	No	No	17
Singh et al.^28^	Yes	No	No	Yes	No	Yes	Yes	No	No	No	14
Zeng et al.^29^	No	Yes	Yes	No	No	Yes	Yes	Yes	No	No	15
Lau, Hagg^30^	No	No	No	Yes	Yes	No	No	No	No	No	12
Chang et al.^31^	Yes	Yes	No	Yes	Yes	Yes	Yes	Yes	Yes	No	18
Yoon, Chung^37^	Yes	Yes	Yes	No	No	Yes	Yes	Yes	Yes	No	17

A qualitative evaluation of the methodology used by these articles was performed according to previous studies.^17^ ^,^ ^18^ The variables being considered for review are listed in Table 1, including corresponding values. Table 2 shows the articles presenting more accurate methodologies.

Each article was given points according to the items evaluated, as can be seen in Table 1. Each item had a maximum score of 2 points if there was a consensus on it, otherwise 1 point was given. This procedure was applied to each article.

Therefore, each article could reach a maximum score of 20 points, thus allowing the study quality to be ranked as follows: ≤ 10 points = low score; > 10 and ≤15 = average score; > 15 and ≤ 18 = moderately high score; and > 18 points = high score.

Table 3 shows the sample characteristics, including the objective of each article, and Table 4 lists the epitomes. In this last table, a study^22^ was excluded because it did not present total measures, but rather growth increments.

**Table 3:** Sample characteristics and objectives of the 16 articles selected for review.

Article	Type of study (T or L)	n	Control group	Malocclusion	Sex %	Ethnicity	Age	Objective
Dhoptkar et al.^19^ (2002)	Transversal	200	No	50 with Class I 50 with Class II div 1 50 with Class II div 2 50 with Class III	50%	Caucasian	Class I: 10.4 yrs Class II div 1: 10.1yrs Class II div 2: 11.1yrs Class III: 10.2 yrs	To compare the skull base flexion to type of malocclusion as follows: Class I to Class II div 1 to Class II div 2 and Class I to Class III
Wilhelm et al.^15^ (2001)	Longitudinal	43	No	22 with Class I 21 with Class II	50%	European	1 month 2 yrs and 14 yrs old	To compare the longitudinal growth of the skull base (size and shape) to Class I and Class II malocclusions)
Mouakeh^21^ (2001)	Transversal	138	Yes; Paired by age, sex and ethnicity	69 with Class III 69 with Class I (control)	66.6% female	Syrian	5 - 12 yrs old Mean 8.4 ± 2 yrs old	To identify morphological characteristics of the craniofacial complex in Syrian patients with Class III malocclusion and compare them to controls
Alexander et al.^22^ (2009)	Longitudinal	103	No	103 with Class III	55 females 48 males	Caucasian	6-7; 7-8; 8-9; 10-11; 11-12; 12-13 13-14; 15-16; 16-17; 17-18 yrs old	To investigate changes in the craniofacial growth in untreated individuals with Class III malocclusion
Ishii et al.^23^ (2001)	Transversal	279	Yes	190 with Class II div 1 89 with Class I (control)	100% females	Japanese	3 groups: Mixed dentition, Delayed mixed dentition, Young permanent dentition	To compare the craniofacial characteristics of Japanese females with Class II division to those of controls
Polat, Kaya^1^ (2007)	Transversal	75	No	25 with Class I 25 with Class II 25 with Class III	CIass I: 13 F, 11 M Class II: 14 F, 11 M Class III: 13 M, 12 F	Not specified (patients treated at the Baskent University in Ankara, Turkey	15.74 yrs ± 4.28	To evaluate the difference in the skull base flexion between dental and skeletal Class I, Class II, and Class III malocclusions
Singh et al.^24^ (1997)	Transversal	142	Yes	73 with Class III 69 with Class I (control)	Approximately the same number of males and females	European/American	7 groups of 5 to 11 yrs	To differentiate the skull base between individuals with Class I malocclusion and those with Class III malocclusions
Johannsdottir et al.^25^ (1999)	Transversal	563	Yes	200 with Class I 363 with Class II	184 males 179 females	Icelanders	5 yrs 7 mo to 7 yrs 8 mo Mean of 6 yrs 7 mo	To describe the craniofacial characteristics of Icelander children aged 6 years old with Class II malocclusion and compared them to Class II malocclusion
Rothstein, Yoon-Tarlie^16^ (2000)	Transversal	613	Yes	278 with normal occlusion 335 with Class II div 1	T1: 47 F and 48 M T2: 47 F and 43 M	Caucasian	3 groups of subjects aged 10, 12 and 14 yrs old ± 6 years	To investigate whether Class II malocclusion is characterized by a poorly developed mandible or by its retropositioning
Ishii et al.^26^ (2002)	Transversal	124	No	Class II div 1 in Japanese females Classe II div 1 in Caucasian females	100% females	49 Japanese and 75 Caucasian females	Japanese: 11 yrs 8 mo British: 11 yrs 11 mo	To help define the craniofacial morphology of the Japanese with Class II malocclusion div 1 and to compare them with Caucasian individuals with the same malocclusion; and to elucidate craniofacial differences between ethnic groups
Kapoor et al.^27^ (2001)	Transversal	100	Yes	50 with Class II 50 with normal occlusion	50%	Indian	9 to 12 yrs old 10.64 yrs old, males 9.8 yrs old, females	To understand and differentiate the skeletal-dental morphology of children with straight terminal plane and those with Class II pattern
Singh et al.^28^ (1998)	Transversal	141	No	72 with Class III, European/American 69 with Class III, Korean	50%	European/American Korean	5 to 11 yrs old	To compare Korean and European/American subjects with Class III malocclusion and provide data on ethnic diversity in individuals with Class III malocclusion
Zeng et al.^29^ (1998)	Transversal	160	No	40 with Class I Chinese; 40 with Class II Chinese (ch) 40 with Class I Swedish 40 with Class II Swedish (sw)	50%	Chinese Swedish	8 to 10 yrs old 9.1 ± 0.9 males (M) ch Class II 9 ± 0.8 females (F) ch Class II 9.4 ± 0.8 M/F ch Class I 9.3±0.9 M sw Class I 9.3 ± 0.8 F sw Class I 8.7±0.8 M sw Class II 8.9±1.0 F sw Class II	To assess craniofacial structures in two ethnic groups and two malocclusions by comparing Chinese and Swedish individuals with Class I and Class II malocclusions
Lau e Hagg^30^ (1999)	Transversal	416	No	105 Chinese with Class II div 1 107 Caucasians with Class II div 1 204 Chinese with normal occlusion	Approx. 50%	Chinese Caucasians	10 to 15 yrs old 11 to 13 yrs old 12 yrs old	To assess the craniofacial pattern of Chinese and Caucasian individuals with Class II div 1 malocclusion and those with normal occlusion; Chinese with Class II x Chinese with normal occlusion; Chinese with Class II div 1 x Caucasians with Class II div 1
Chang et al.^31^ (2005)	Transversal	200	Yes	100 with Class III 100 with normal occlusion (control)	50%	No specification (radiographs from the Kaohsiung University, Taiwan)	9.4 to 11.5 yrs old	To compare morphological characteristics of the skull base in children with malocclusion Class III with normal children and provide database for subsequent Class III studies
Yoon, Chung^37^ (2015)	Longitudinal	46	Yes	25 with Class I 21 with Class II	100% females	Caucasian	ages 9, 14, and 18	To investigate and compare the craniofacial growth of untreated Class I and Class II girls from ages 9 to 18 years

**Table 4:** Epitome of the articles selected for review.

Article	Flexion (NSBa or NSAr)	Anterior length (N-S)	Posterior length (S-Ba or S-Ar)	Conclusion
Dhopatkar et al.^19^ (2002)	NSBa: non-significant / NSAr: more significant for Class II div 1 malocclusion than for Class I malocclusion. No difference was observed in the latter	N-S: greater for Class II div 1 and 2 than in Class I malocclusion Class I and Class II malocclusions no significant	S-Ba: greater in Class II div 1 and 2; than in Class I Class I and Class III malocclusions no significant S-Ar was not studied	The skull base angle itself has no key influence on the development of malocclusions
Wilhelm et al.^15^ (2001)	NSBa: not so obtuse in patients with Class II malocclusion, flexion decreases from 1 month to 2 years and 14 years. NSAr was not studied	N-S: no difference was found between Class I and Class II malocclusions	S-Ba: no difference between malocclusions S-Ar was not used	Growth in skull base is similar to that in Class I and Class II malocclusions
Mouakeh^21^ (2001)	NSBa was not studied; NSAr was smaller in Class III than in normal occlusion	N-S smaller in Class III than in normal occlusion	S-Ba was not studied S-Ar smaller in Class III than in normal occlusion	Flexion, anterior and posterior lengths are significantly smaller in Class III malocclusions
Alexander et al.^22^ (2009)	No measurement proposed was used	N-S increased yearly in all age groups studied, 1 mm in boys and less than 1 mm in girls	S-Ba not studied S-Ar not studied	No measurement proposed was used
Ishii et al.^23^ (2001)	NSBa was not studied NSAr: not significant between age groups for Class II div 1 malocclusion Not evaluated for Class I malocclusion.	N-S smaller in Japanese young permanent dentition with Class II div I malocclusion than in those with normal occlusion	S-Ba not studied S-Ar: not significant between age groups for Class I div 1 malocclusion Not evaluated between Class II div 1 malocclusion and Class I malocclusion	Class II div 1 malocclusion had an increase in facial angle in association with a shortened mandibular ramus, compared to Class I malocclusion
Polat, Kaya^1^ (2007)	NSBa not significant NSAr not significant	N-S not significant	SBa not different S-Ar not significant	No difference was found in angle and dimensions of the skull base for Class I, II and III malocclusions
Singh et al.^24^ (1997)	NSBa was more acute in Class III at 8 to 9 yrs old NSAr was not different between Class I and Class III malocclusions at 5-11 yrs old despite tending to be acute	N-S smaller in Class III than Class I malocclusions at 8 and 9 yrs old	S-Ba: no difference between age groups S-Ar not studied	Skull base angle tending to be more acute in Class III malocclusion than in normal occlusion
Johannsdottir et al.^25^ (1999)	NSBa not significant between men and women, being more obtuse in Class II than in Class I malocclusions NSAr not significant between men and women, being more obtuse in Class II than in Class I malocclusions	N-S was greater in Class II than in Class I malocclusion, dimorphism; SN smaller in women; SN was significant	S-Ba and S-Ar smaller in women	Dimorphism was found at the skull base at 6 yrs old in Class II malocclusion
Rothstein, Yoon-Tarlie^16^ (2000)	NSBa greater in Class II div 1 malocclusion for girls at 10, 12, 14 yrs old (no difference); and boys at 10, 12, 14 yrs old. NSAr not studied	N-S was significantly greater in Class II div 1 malocclusions for girls at 10, 12, 14 yrs old and for boys at 14 yrs old compared to Caucasians with normal occlusion	S-Ba: no significant difference found in girls Significant difference was found in boys at 10 and 12 yrs old only S-Ar not studied	Anterior base is more protrusive in Class II malocclusions, with length being excessive at the skull base, maxillary and frontal sinuses increased (may contribute to Class II malocclusion increase) Skull base flexion does not contribute to the retruded positioning of the mandible
Ishii et al.^26^ (2002)	NSBa not studied NSAr smaller in Japanese than in Caucasian women despite the lack of significant difference	N-S was significantly smaller in Japanese than in Caucasian women with Class II div 1 malocclusions	S-Ba not studied S-Ar: no significant difference	Caucasian women have a more significantly longer anterior skull base
Kapoor et al.^27^ (2001)	NSBa not studied NSAr: no significant gender difference was found despite being smaller in women. More acute for distal malocclusions	N-S not studied	S-Ba not studied S-Ar not studied	NSAr is one of the factors indicating a Class II pattern in association with a distal mandibular positioning and skull base rotation
Singh et al.^28^ (1998)	NSBa and NSAr are more acute in European/American individuals than in Korean ones with Class III malocclusion	N-S greater in European/American individuals	S-Ba and S-Ar greater in European/American individuals	For European/American individuals with Class III malocclusions, the craniofacial morphology is affected by an orthocephalization of the skull base, exacerbated by prominent mandible and symphysis morphology
Zeng et al.^29^ (1998)	NSBa: no significant difference was found between Swedish and Chinese individuals with Class I and Class II malocclusions NSAr not studied.	N-S greater in Chinese individuals with Class II malocclusions than in Swedish ones, despite being smaller in Chinese individuals with Class I malocclusion, compared to the Swedish ones S-Ar not studied	S-Ba greater in Chinese individuals with Class II malocclusions than in Swedish ones No significant difference was found in Class I malocclusion regarding both ethnic groups S-Ar not studied	Anterior and posterior lengths of the skull base were greater in Chinese than in Swedish individuals
Lau, Hagg^30^ (1999)	NSBa not studied NSAr: no significant gender difference in Chinese individuals with Class II div 1; however, this angle was smaller when Class II div 1 malocclusion was compared to normal occlusion	S-N not studied	S-Ba not studied S-Ar not studied	No dimorphism was found between Chinese individuals with Class II div 1 malocclusion Skull base angle is more acute in Chinese individuals with Class II div 1 malocclusion than in those with normal occlusion
Chang et al.^31^ (2005)	NSBa not significant NSAr: more acute in Chinese individuals with Class III malocclusion	N-S smaller in Class III malocclusion than in normal occlusion	S-Ba smaller in Class III malocclusion, but with no significant difference S-Ar smaller in Class III malocclusion than in normal occlusion	The decreased flexion and length in Class III malocclusion may be related to the aetiology of this type of malocclusion
Yoon, Chung^37^ (2015)	NSAr ( Saddle angle) not significant	N-S not significant	S-Ba not studied S-Ar not studied	In general, the Class I and Class II groups showed similar skeletal growth

### Age

Seven studies have investigated the growth of the skull base by comparing the data obtained from different age groups^16^ ^,^ ^20^ ^,^ ^22^ ^,^ ^23^ ^,^ ^24^ ^,^ ^28^ ^,^ ^37^ with three^22^ ^,^ ^24^ ^,^ ^28^ of them using samples of individuals with Class III malocclusion and whose age ranged from 5 to 18 years old. These individuals were found^22^ to have a yearly increment in the length of the anterior skull base in all age groups (6 to 18 years old), which was smaller than 1 mm for women and approximately equal to 1 mm for men, similar to the growth estimated for individuals with Class I malocclusion. In another study^24^ comparing Class III malocclusion to Class I malocclusion in individuals aged 5 to 11 years old, it was found that NSBa angle was more acute in the latter at ages of 5, 8, and 9 years old, and that NSAr angle had the same trend; but no statistically significant difference was found at 8 years old. Researchers^23^ have reported that the skull base of Japanese individuals with Class II division 1 malocclusion had a significantly smaller anterior length at 10 years and 10 months to 15 years and 10 months old compared to other age groups, involving individuals with same malocclusion and race (Group 1: 7 years and 6 months to 13 years and 6 months old; and Group 2: 9 years and 1 month to 13 years and 6 months old). On the other hand, another study^29^ compared individuals with Class II division 1 malocclusion to controls and found that the length of the anterior skull base was statistically greater in all six age groups studied (females aged 10, 12, 14 years old, and males aged 10, 12, 14 years old). Two studies^20^ ^,^ ^28^ had samples with different age groups, but it was not possible to use all data because the first study^20^ had compared age groups of 1 month, 2 years and 14 years old, whereas the second study^28^ had assessed neither flexion nor the skull base length at different age groups. For Yoon and Chung,^37^ which compared Class I with Class II at three different ages (9, 14 and 18 years - all female), no difference was observed in the flexion or length of the anterior skull base.

### Ethnic group

Three studies have assessed different ethnic groups,^26^ ^,^ ^28^ ^,^ ^29^ with Asian being compared to Caucasian individuals in most cases, and significant differences being found. The length of the anterior skull base was found to be greater for Japanese than for Caucasian females.^26^ However, a study^28^ reported a greater anterior skull base as well as a more acute NSAr angle in European/American than in Korean individuals. Similarly, with regard to the length of the anterior skull base, a study^29^ showed this measurement was smaller in Chinese than in Swedish individuals for both types of malocclusions (Class I and Class II), with the posterior length (S-Ba) being also smaller in Chinese individuals with Class II malocclusion. Another study^24^ evaluated two ethnic groups but no comparison was possible because of the lack of data on skull base structures regarding Caucasian individuals.

### Dimorphism

Six studies have evaluated the relationship between sexual dimorphism and development of the skull base,^22^ ^,^ ^25^ ^,^ ^27^ ^,^ ^30^ ^,^ ^16^ ^,^ ^29^ but no significant difference was found in the angular measurements. However, one study^25^ showed that linear measurements (S-N, S-Ar and S-Ba) were significantly greater in Icelander male children compared to female ones with Class I and Class II malocclusions.

### Malocclusion differences

Of the 16 articles selected, only one has not compared flexion or length of the skull base to some type of malocclusion.^22^ Two studies^1^ ^,^ ^19^ compared flexion or length of the skull base in individuals with Class I, Class II and Class III malocclusions. One of these studies^19^ found greater angular measurements for the skull base in Class II division 1 malocclusion, compared to Class I malocclusion. In this same study, no difference was found between Class I malocclusion and other ones, regarding such angular measurements, whereas the length of the anterior and posterior skull base were greater in the cases of Class II malocclusion. However, the other study^1^ reported significant differences in the skull base length between the three types of malocclusion. Studies^21^ ^,^ ^24^ ^,^ ^31^ have also compared Class III malocclusion to normal occlusion, whereas another one^28^ compared this condition between Caucasian and Korean individuals. All these studies found similar results, with the length of the anterior skull base being smaller in Korean individuals with Class III malocclusion and skull base flexion tending to be more acute in Caucasian individuals with the same condition.

Among these seven studies^16^ ^,^ ^20^ ^,^ ^23^ ^,^ ^25^ ^,^ ^27^ ^,^ ^30^ ^,^ ^37^ comparing Class II malocclusion to Class I malocclusion, similar results have been reported. For example, individuals with Class II malocclusion had greater anterior and posterior lengths and more obtuse angular measurements regarding the skull base. However, one study^23^ reported a smaller length of the skull base in Japanese girls with Class I malocclusion presenting permanent dentition, whereas another^30^ found no significant differences in the skull base flexion compared to normal occlusion. In the study, the compared groups (Class I and Class II) showed no differences in the length of the anterior skull base and flexion of the cranial base.

## DISCUSSION

The skull base not only supports and protects the brain but also articulates the cranium with vertebral column, maxilla and mandible. One of its key functions has to do with adaptation and protection, including a shock-absorbing area between brain, face and pharyngeal region, whose growths occur differently. The growth of the skull base occurs by means of a complex balance between sutural growth, prolongation of synchondroses, extensive cortical sliding, and remodelling. This combination allows an increase in the differential growth between base and vault of the skull, expansion of the contours of the various endo-cranial fossae, maintenance of vessel and nerve pathways, and prolongation of the processes, such as hypophysis. Prolongation of the skull base occurs with the growth of synchondroses and direct cortical growth. The cortical sliding of the skull floor produces several degrees of growth movement at different regions, usually towards the ecto-cranial direction, with apposition proportional to the external surface.^5^

Enlow^32^ has shown that maxilla growth is influenced by the skull base, which in turn, is influenced by the growth of the brain. The mandible, because of its distant positioning, acts more independently, despite being articulated with glenoid fossa, thus being a potential factor influencing the skull base.

To better understand the cephalometric aspects, the skull base is divided into anterior and posterior lengths, the former extending anteriorly from sella turcica (S) to nasofrontal suture (N), and the latter extending from sella turcica to the anterior edge of the foramen magnum, defined as Ba.^21^ There is a consensus that the length of the anterior skull base corresponds to the linear N-S distance, but the same cannot be said about the posterior region, which corresponds to either S-Ba or S-Ar linear distances.^19^

Björk^2^ supported the use of the latter, as it is more easily visualized, with most studies using this measurement. Verjanne and Koski^33^ suggested the use of Ba to measure the skull base angle as they considered the S-Ar measurement too distant; Kerr and Adams^34^ used Ba to measure the skull base angle. Bhatia and Leighton^35^ used N-S-Ba, N-S-Art as well as S-Ba and S-Art, and found similar measurements.

According to the other authors,^19^ the skull base follows a neural (anterior region) and somatic (posterior region) growth pattern despite its cartilaginous origin (chondrocranium). After birth, especially in the early childhood, the growth of the anterior portion occurs mainly due to the increase in frontal sinus and remodelling of the nasal region, whereas the growth of the posterior region is related to the interstitial growth occurring in the spheno-occipital synchondrosis.

The two segments of the base of the skull form an flexion of 130-135 degrees at the angle formed at the Sela point (center of the sella turcica). This angle (NSBa) has approximately 142 degrees at birth, but decreases to 130 degrees at 5 years old.^19^ From 5 to 15 years old, the skull base angle is relatively stable.^23^ Other studies suggest that there are no differences in this angle of the skull base during childhood, puberty and adult phase.^1^ ^,^ ^23^

There is evidence showing that the skull base angle (N-S-Ba) is greater in Class II division 1 malocclusion than in Class I malocclusion or normal occlusion, with this angle not differing between Class II division 2 and Class I malocclusions.^19^ In addition, studies comparing Class II malocclusions to normal occlusion or Class I malocclusion^16^ ^,^ ^20^ ^,^ ^23^ ^,^ ^25^ ^,^ ^27^ ^,^ ^30^ ^,^ ^37^ found similar results.^16^ ^,^ ^25^ ^,^ ^27^ However, two studies^30^ ^,^ ^37^ compared such malocclusions and reported a smaller flexion of the skull base in individuals with Class II malocclusion. This can be explained by the fact that the posterior region of the skull base (S-Ba) forming the S-N-Ba angle might be anteriorly or posteriorly inclined, whereas the anterior region (S-N) might also be inclined anteriorly upwards or downwards, thus causing a variation in either S or N points vertically.^36^ Therefore, variable lengths of the anterior and posterior regions of the skull base can compensate any cranial flexion, that is, a posterior acute angle anteriorly positioned in relation to the mandible can neutralize the cranial flexion through the greater posterior length, thus positioning Ba and mandible posteriorly and vice-versa.^20^ However, the skull base length was not assessed.^30^ Despite these findings, the skull base flexion is thought to have no influence on malocclusions,^1^ ^,^ ^19^ whereas there is no consensus among other studies regarding this issue.^21^ ^,^ ^23^ ^-^ ^27^ ^,^ ^30^ ^-^ ^31^

With regard to the Class III malocclusion, it has been reported that linear and angular measurements of the skull base are smaller when compared to other types of malocclusion.^1^ These findings are corroborated by some studies,^21^ ^,^ ^31^ although one study^1^ had found smaller angular measurements for Class III malocclusions, despite not being significant.

Asian individuals present a smaller anterior length and a more obtuse angle of the skull base, compared to Caucasians, with this finding comprising the both ethnic and morphological characteristics, according to some authors.^23^ ^,^ ^26^ ^,^ ^28^

## CONCLUSIONS

After evaluating all these articles selected for the present systematic review, one can state the following:

1. The skull base angle itself does not seem to play a key role in the development of malocclusions.
2. The skull base angle is relatively stable at the ages of 5 to 15 years old.
3. A more obtuse skull base flexion, in association or not with a greater length of the anterior skull base, can contribute to the development of Class II division 1 malocclusion. A more acute skull base flexion can contribute to a more anterior positioning of the mandible and to development of Class III malocclusion as well.
